# Clinical characteristics and prevalence of adolescent self-harm before, during and after the COVID-19 pandemic: retrospective cross-sectional database study

**DOI:** 10.1192/bjo.2025.4

**Published:** 2025-03-10

**Authors:** Patricia Zavaleta-Ramírez, Marcos Rosetti, Lilia Albores-Gallo, Emmanuel Sarmiento-Hernández

**Affiliations:** Juan N. Navarro Children’s Psychiatric Hospital, Psychiatric Care Services, National Commission on Mental Health and Addictions (CONASAMA), Mexico City, Mexico; Institute of Biomedical Research (IIBO), National Autonomous University of Mexico (UNAM), Mexico City, Mexico; Dr Ramón de la Fuente Muñiz National Institute of Psychiatry, Mexico City, Mexico

**Keywords:** Cutting, depression, middle-income country, Mexico, post-pandemic

## Abstract

**Background:**

Evidence indicates that the COVID-19 pandemic has led to an increase in self-harm among adolescents; however, little is known about the trends in prevalence after the end of the pandemic.

**Aims:**

This retrospective cross-sectional database study evaluates changes in the prevalence and clinical characteristics of self-harm among adolescents who sought attention from the emergency service of the largest children’s psychiatric hospital in Mexico before, during and after the COVID-19 pandemic.

**Method:**

After comparing the clinical characteristics of self-harm between the three periods, we calculated the monthly prevalence of self-harm among patients (*n* = 3520) visiting the hospital’s emergency psychiatric services over a period of 58 months. Using joinpoint regression, we evaluated temporal trends in self-harm prevalence.

**Results:**

Affective disorders and hitting as a method of self-harm were more frequent during and after the pandemic in comparison with the pre-pandemic period. The prevalence of self-harm diminished from March 2019 to March 2023, a trend followed by an increase coinciding with the end of the pandemic and the return to normal activities.

**Conclusions:**

The significant increase in prevalence observed after the end of the pandemic suggests a long-term impact on mental health of adolescents. This underscores the need for monitoring this population during post-pandemic years to provide timely interventions.

Self-harm refers to the act of intentionally causing harm to oneself, regardless of type, motive or suicidal intent.^1^ The lifetime prevalence of self-harm in community samples is estimated to be 16.9%,^2^ with higher rates reported for non-suicidal self-injury in clinical samples.^3,4^ Specifically, the pooled prevalence of non-suicidal self-injury among adolescents during the COVID-19 pandemic was 15.70%.^5^ Self-harm is a risk factor for subsequent suicidal behaviour.^6^ Individuals who engage in self-harm often experience barriers to accessing mental healthcare, such as long waiting times^7^ or perceived stigma,^8^ and approximately 50% do not seek medical attention.^9^

## Self harm and the COVID-19 pandemic

Evidence regarding the impact of the COVID-19 pandemic on self-harm, including suicide attempts, is mixed. Early reports showed a decrease in hospital visits during lockdown,^10–14^ whereas later studies with longer follow-up periods reported changes in case prevalence.^15–23^ For example, in the USA, Mayne et al^16^ found an increase from 6.1 to 7.1% when screening for suicide risk in adolescents in primary care between October and December 2020, with a 34% relative increase in suicidal ideation among females. Similarly, Hiscock et al^18^ observed a 12.7 percentage point increase in self-harm presentations in Australia during lockdown and Wong et al’s study,^22^ which analysed patients’ records from 32 countries, including Mexico, further highlighted a doubling of self-harm rates between March and April 2021.

## The case of Mexico

Recently, a meta-analysis of 42 studies across 18 countries concluded that although suicide attempts and suicidal ideation significantly increased, only a moderate rise in self-harm was observed in children and adolescents presenting to emergency departments during the COVID-19 pandemic compared with pre-pandemic rates.^23^ However, this meta-analysis lacked evidence from Latin America. The Mexican nationwide household survey found no increase in suicide attempts among adolescents during the first 5 months of the COVID-19 pandemic compared with the previous year.^24^ Further studies in this region are critical, given that the return to in-person classes in Mexico occurred later than in North America or Europe and studies beyond the second year of the pandemic are scarce.

In Mexico, a lockdown took place from April to May 2020, consisting of a nationwide cessation of non-essential activities, followed by a stay-at-home advisory. In-person academic activities for children and adolescents were suspended until August 2021, transitioning to online classes and reduced schedules during the 2020–2021 school year.^25^ Additionally, vaccination campaigns started later than in other North American countries, with adolescents being the last to be vaccinated, further delaying their return to social activities and peer interactions. Furthermore, Mexico also experienced one of the highest rates of children losing primary or secondary caregivers (5.1 per 1000 children).^26^ These circumstances likely represented chronic stress for Mexican youth, potentially exacerbating mental health problems compared with regions where the return to ‘normal’ was more streamlined.

Given the documented negative impact of the pandemic on youth in community samples,^10^ it is essential to assess long-term consequences in clinical samples of children and adolescents following their return to normal routines. This study aims to examine self-harm in Mexican adolescents presenting to the emergency department of a child psychiatric hospital. We analysed monthly self-harm prevalence over 58 months (March 2019 to December 2023), covering pre-, post- and pandemic periods. Additionally, we provide clinical details regarding self-harm characteristics and psychiatric diagnoses.

## Method

### Data collection

The study was conducted at the Juan N. Navarro Children’s Psychiatric Hospital in Mexico City. The hospital provides psychiatric care for children and adolescents with mental disorders, suicide attempts and non-suicidal self-harm. It serves children and adolescents from paediatric hospitals in Mexico City, the surrounding metropolitan area and neighbouring states, covering a population of approximately two million minors. Patients may arrive through referrals from general hospitals or as walk-ins. At the hospital, the first point of contact is a psychiatrist, who conducts a 10 min triage to determine whether the patient requires emergency psychiatric evaluation, out-patient services, referral to another unit or is discharged. During the pandemic lockdown (April and May 2020), services were limited to emergency presentations and the psychiatric ward.

The emergency psychiatric department follows a standardised procedure for collecting self-harm data. Child and adolescent psychiatry residents perform diagnostic assessments through clinical interviews lasting 40–60 min. When self-harm is identified, clinicians collect sociodemographic and clinical information using a violence and injury reporting form. These data are stored in an epidemiological reporting database.

For this study, de-identified data were extracted and associated with a unique alphanumeric code for each patient. The variables included date of visit, age, gender, academic level, literacy, location (place of occurrence) and method of self-harm, anatomical area, consequences (emotional or physical), clinical management (admission or referral) and main psychiatric diagnosis coded according to ICD-10.

From the hospital records we obtained the total number of patients visiting the emergency department to calculate overall prevalence of self-harm. We used a unique code for each patient to remove repeated cases either (a) per period (pre-, during and post-pandemic) or (b) per month. We included children and adolescents who had engaged in self-harm behaviour regardless of motive, as defined by the National Institute of Clinical Excellence,^1^ and we excluded cases where injuries were inflicted by third parties (e.g. abuse or assault). The study period spanned March 2019 to December 2023.

### Statistical analysis

We report frequencies and percentages for sociodemographic and clinical characteristics. We used chi-squared tests to compare the prevalence of self-harm characteristics and main diagnoses across the pre-pandemic, pandemic and post-pandemic periods. Categories with low statistical power because of small sample sizes were omitted from tables. Missing data accounted for less than 0.001%; cases with missing cells were excluded on a variable-by-variable basis.

Monthly self-harm prevalence was calculated by dividing the number of unique self-harm cases by the total number of unique emergency department visits. We used joinpoint regression, a statistical technique that allows identification of trend change points (i.e. decreasing versus increasing periods), to evaluate trends in monthly self-harm prevalence during the whole 58-month period.^27^ We used the joinpoint() function from the nih.joinpoint package (https://github.com/DanChaltiel/nih.joinpoint). We specified six maximum joinpoints, which is recommended for data-sets larger than 27 points. A larger number of joinpoints is possible (9 maximum) and could improve the resolution in terms of the trend points detected, although computing time will increase exponentially. We specified three cores in the processor to parallelise computation; more cores reduce computing time. We stratified prevalence trends by gender to account for potential differences, as females comprised the majority of the sample. All analyses were conducted in R version 4.3.2^28^ and statistical significance was set at *P* < 0.05.

### Ethical statement

The authors assert that all procedures contributing to this work comply with the ethical standards of the relevant national and institutional committees on human experimentation and with the Helsinki Declaration of 1975, as revised in 2008. The study was approved by the ethics board of the Juan N. Navarro Children’s Psychiatric Hospital (num. HPIJNN-CEI-DA-010-2021). De-identified data for the study were extracted from the psychiatric emergency department general database. Written or verbal informed consent from the participants’ legal guardian/next of kin was not required for inclusion in this study, in accordance with current legislation and institutional requirements.

## Results

Table 1 shows the sociodemographic characteristics of paediatric patients with self-harm visiting the emergency services before (March 2019 to February 2020), during (March 2020 to May 2023) and after (June 2023 to December 2023) the COVID-19 pandemic. For all three periods, patients’ mean age was around 14 years, most were female and almost all were enrolled in school. Table 2 shows self-harm characteristics for each of the three time periods. Before, during and after the pandemic self-harm took place predominantly at home (88.3%).

**Table 1:** Demographic characteristics of patients presenting before, during and after the COVID-19 pandemic

	Pre-pandemic (*n* = 931)	During pandemic (*n* = 2130)	Post-pandemic (*n* = 459)
Age, years: mean (s.d.)	14.5 (1.80)	14.5 (1.77)	14.7 (1.83)
Gender (female), *n* (%)	637 (68.42)	1600 (75.12)	337 (75.56)
Can read and write (yes), *n* (%)	918 (98.6)	2002 (93.99)	440 (98.65)
Academic level, *n* (%)			
University	0 (0)	3 (0.14)	1 (0.22)
High school	296 (31.79)	754 (35.4)	171 (38.34)
Middle school	542 (58.22)	1103 (51.78)	220 (49.33)
Elementary	85 (9.13)	174 (8.17)	49 (10.99)
None	8 (0.86)	96 (4.51)	5 (1.12)
Pregnant (yes), *n* (%)	0 (0)	9 (0.56)	0 (0)
Physical disability (yes), *n* (%)	4 (0.43)	33 (1.54)	24 (0.52)

**Table 2:** Clinical characteristics of self-harm by periods of the COVID-19 pandemic^a^

	Pre-pandemic, *n* (%)	During pandemic, *n* (%)	Post-pandemic, *n* (%)	During versus pre-pandemic	Post-pandemic versus during	Pre- versus post-pandemic
				Adjusted *P*-values^b^		
Place of occurrence						
Home	844 (90.66)	1874 (87.98)	390 (87.44)	0.108	1	0.249
School	37 (3.97)	50 (2.35)	13 (2.91)	0.054	1	1
Foster care	19 (2.04)	48 (2.25)	7 (1.57)	1	1	1
Self-harm method						
Cutting with sharp object	531 (57.04)	1153 (54.13)	256 (57.02)	0.444	0.864	1
Poisoning	272 (29.22)	539 (25.31)	107 (23.83)	0.081	1	0.126
Hitting or kicking oneself	29 (3.11)	187 (8.78)	38 (8.46)	**<0.001**	1	**<0.001**
Hanging	35 (3.76)	66 (3.1)	12 (2.67)	1	1	1
Hitting or kicking the floor or wall	20 (2.15)	39 (1.83)	7 (1.56)	1	1	1
Asphyxia	21 (2.26)	39 (1.83)	3 (0.67)	1	0.354	0.174
Anatomical area injured						
Upper limbs	514 (54.56)	1142 (53.62)	258 (57.08)	1	0.591	1
Face, neck or head	89 (9.45)	97 (4.55)	17 (3.76)	**<0.001**	1	**<0.001**
Lower limbs	20 (2.12)	88 (4.13)	19 (4.2)	**0.021**	1	0.126
Multiple areas	16 (1.7)	41 (1.92)	13 (2.88)	1	0.81	0.642
Abdomen	11 (1.17)	32 (1.5)	6 (1.33)	1	1	1
Consequence of self-injury						
Skin laceration	345 (36.82)	960 (45.07)	153 (34.3)	**<0.001**	**<0.001**	1
Emotional discomfort	267 (28.5)	761 (35.73)	163 (36.55)	**<0.001**	1	**0.009**
Scars	90 (9.61)	146 (6.85)	63 (14.13)	**0.03**	**<0.001**	**0.048**
Wound	66 (7.04)	92 (4.32)	35 (7.85)	**0.006**	**0.009**	1
Contusion	20 (2.13)	36 (1.69)	8 (1.79)	1	1	1
Clinical management						
Referral to different medical unit	259 (27.82)	867 (40.70)	204 (45.74)	<0.001	0.168	**<0.001**
Referral to out-patient management	201 (21.59)	907 (42.58)	155 (34.75)	**<0.001**	**0.009**	**<0.001**
Hospital admission	381 (40.92)	227 (10.66)	40 (8.97)	**<0.001**	1	**<0.001**
Home	74 (7.95)	109 (5.12)	43 (9.64)	**0.009**	**<0.001**	1
Psychiatric diagnosis (ICD-10)						
Affective disorder	583 (62.62)	1541 (70.21)	336 (74.5)	**<0.001**	0.228	**<0.001**
Anxiety disorder	92 (9.88)	235 (10.71)	32 (7.1)	1	0.078	0.33
ADHD	52 (5.59)	76 (3.46)	20 (4.43)	**0.024**	1	1
Conduct disorder	38 (4.08)	83 (3.78)	14 (3.1)	1	1	1
Personality disorders	36 (3.87)	32 (1.46)	15 (3.33)	**<0.001**	**0.033**	1
Substance use disorder	32 (3.44)	42 (1.91)	13 (2.88)	**0.045**	0.771	1
Psychotic disorder	20 (2.15)	13 (0.59)	1 (0.22)	**<0.001**	1	**0.036**
Neurodevelopmental disorder	26 (2.79)	24 (1.09)	4 (0.89)	**0.003**	1	0.111
Mental disorder due to brain damage	39 (4.19)	29 (1.32)	0 (0)	**<0.001**	0.081	**<0.001**

ADHD, attention-deficit hyperactivity disorder.

a. Percentages do not always add up to 100% as we removed categories with small frequencies (and small statistical power) for clarity.

b. These three columns provide the multiple comparison-adjusted results of chi-squared tests between the time periods. *P*-values <0.05 are marked in bold.

Cutting was the main method of self-harm (55.11%), followed by poisoning (26.07%), in all three periods. Hitting was significantly more frequent during (8.78%) and after (8.46%) the pandemic in comparison with the pre-pandemic period (3.11%). Upper limbs were the anatomical area most frequently injured (54.37%), followed by the face, neck or head (5.76%). Injuries to the face, neck or head were significantly less frequent during (4.55%) and after (3.76%) the pandemic in comparison with the pre-pandemic period (9.45%), whereas injuries to the lower limbs were significantly more frequent during (4.13%) than before the pandemic (2.12%). A mixed pattern was observed for consequences of self-injury, with some being more frequent during the pandemic (skin laceration: 45.07%; emotional discomfort: 35.73%), but others being less frequent (scars: 6.85%; wounds: 4.32%). Post-pandemic, the frequencies of some self-injury consequences returned to pre-pandemic levels (skin laceration – pre: 36.82%, post: 34.36%; wounds – pre: 7.04%, post: 7.85%), whereas others remained high (scars – pre: 9.61%, post: 14.13%; emotional discomfort – pre: 28.5%, post: 36.55%).

The most frequent clinical management decision involved referring the patient to out-patient services (35.9%) or a different medical unit (31.8%). Referrals were affected by the pandemic: patients were sent home more frequently after the pandemic (post: 9.64, during: 5.12%), referral to out-patient management was more frequent during (42.6%) and after the pandemic (post: 34.75%, pre: 21.59%) and hospital admissions were less frequent in the same periods (pre: 40.92%; during: 10.66%; post: 8.97%).

Finally, the diagnostic profiles of the patients show a high prevalence of affective disorders (70%), anxiety (10.2%) and attention-deficit hyperactivity disorder (ADHD) (4.2%). Affective disorders were more frequent during (70.21%) and post-pandemic (74.5% *v*. pre: 62.62%), anxiety was similar during (10.71%) and pre-pandemic (9.88% *v*. post: 7.1%) and ADHD, personality, psychotic and neurodevelopmental disorders were less frequent during the pandemic compared with pre-pandemic (Table 2).

**Fig. 1:**
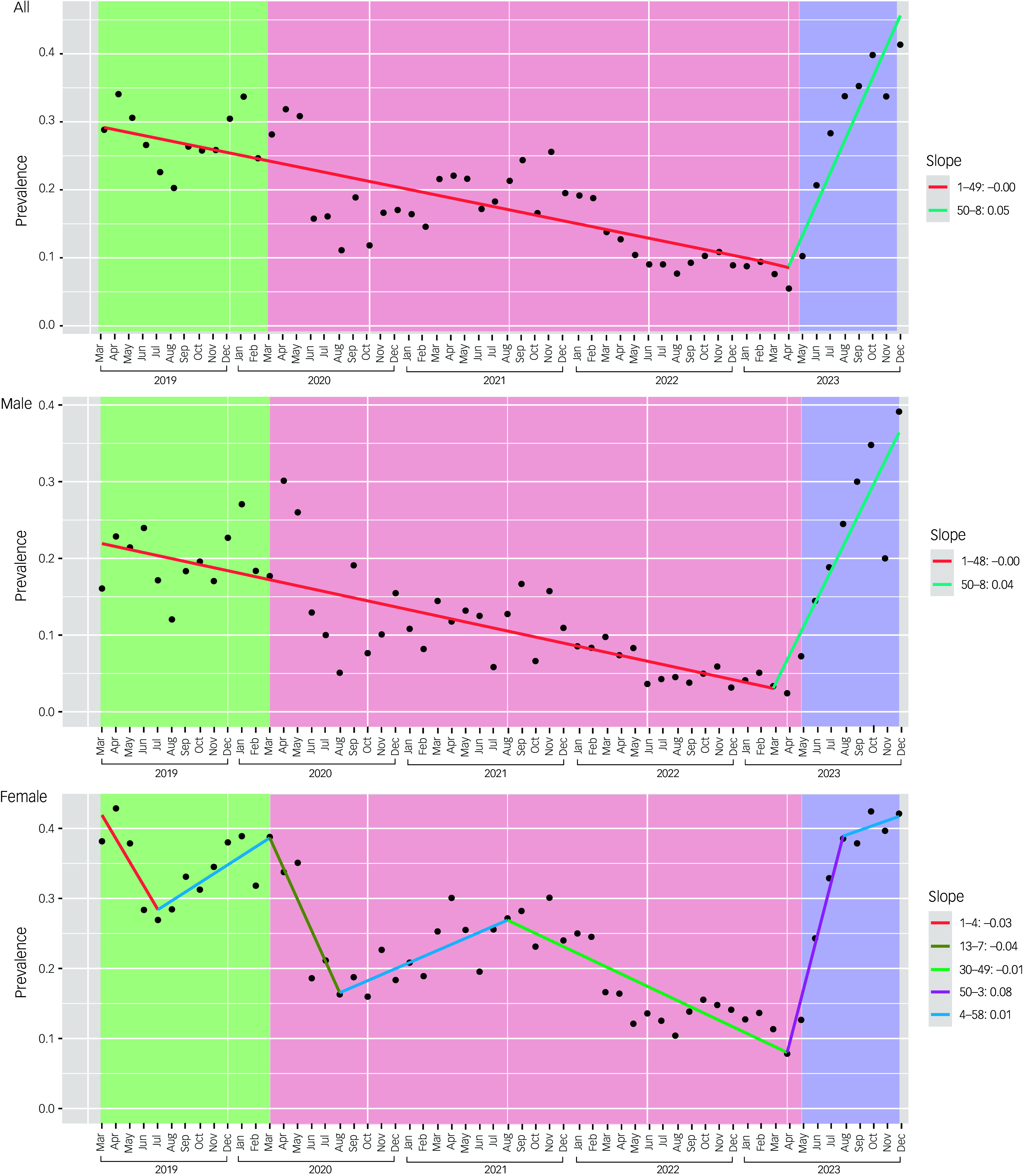
Trends in prevalence of self-harm among patients presenting to the emergency service of a children’s psychiatric hospital in Mexico City from March 2019 to December 2023. Black circles represent observed rates; lines show joinpoint regressions; background shading indicates the three time periods: pre-COVID-19 pandemic (green), during the pandemic (pink) and post-pandemic (blue).

Table 3 shows the slopes of the linear trends in self-harm prevalence produced by the joinpoint regression. The general trend for the full data-set shows two significant inflection points: a negative trend from March 2019 to March 2023 (slope −0.004 ± 0) and a positive one from March to December 2023 (slope 0.046 ± 0.007), which coincides with the post-pandemic period. Male patients mimic this pattern, albeit with sparser data. In contrast, for female patients, the analysis found six inflection points. The slopes of the joinpoints found in the pre- and pandemic periods show a general downward trend (slope_seg0_ −0.034 ± 0.017, slope_seg2_ −0.044 ± 0.017, slope_seg4_ −0.009 ± 0.002), with small positive trends in segments 1 (Jul 2019 to Mar 2020, slope_seg1_ 0.013 ± 0.007), segment 3 (Aug 2020 to Aug 2021, slope_seg3_ 0.009 ± 0.004) and segment 5 (Apr to Jul 2023, slope_seg5_ 0.077 ± 0.026). The last segment shows a large positive trend that also coincides with the end of the pandemic. All trends are presented in Fig. 1.

**Table 3:** Summary of each of the linear segments detected by the joinpoint regression

	Segment	Time period	Intercept (s.e.)	*P*	Slope (s.e.)	*P*
All						
(d.f. = 54, SSE = 0.124, MSE = 0.002)	0	Mar 19 to Mar 23	0.296 (0.014)	**<0.001**	−0.004 (0)	**<0.001**
	1	Mar 23 to Dec 23	−2.221 (0.4)	**<0.001**	0.046 (0.007)	**<0.001**
Male						
(d.f. = 54, SSE = 0.118, MSE = 0.002)	0	Mar 19 to Mar 23	0.223 (0.014)	**<0.001**	−0.004 (0)	**<0.001**
	1	Mar 23 to Dec 23	−1.785 (0.328)	**<0.001**	0.037 (0.006)	**<0.001**
Female						
(d.f. = 44, SSE = 0.054, MSE = 0.001)	0	Mar to Jul 19	0.453 (0.045)	**<0.001**	−0.034 (0.017)	**0.048**
	1	Jul 19 to Mar 20	0.22 (0.064)	**0.002**	0.013 (0.007)	0.074
	2	Mar to Aug 20	0.962 (0.257)	**<0.001**	−0.044 (0.017)	**0.011**
	3	Aug 20 to Aug 21	0.01 (0.085)	0.909	0.009 (0.004)	**0.019**
	4	Aug 21 to Apr 23	0.552 (0.062)	**<0.001**	−0.009 (0.002)	**<0.001**
	5	Apr to Jul 23	−3.782 (1.358)	**0.008**	0.077 (0.026)	**0.005**
	6	Jul to Dec 23	0.009 (0.933)	0.992	0.007 (0.017)	0.672

MSE, mean squared error; SSE, sum of squared errors. *P*-values <0.05 are marked in bold.

## Discussion

The current study reports changes in the prevalence and clinical characteristics of patients with self-harm who presented to a children’s psychiatric hospital emergency department in Mexico City before, during and after the COVID-19 pandemic. We observed (a) changes in clinical characteristics during these periods and (b) a downward trend in the prevalence of self-harm throughout the pandemic, followed by an upward trend that coincided with its end.

### Sociodemographic data

The sociodemographic data in our study are consistent with those reported in the literature, indicating that self-harm is more prevalent among females. The mean age at presentation with self-harm aligns with findings from other clinical studies in Mexico^29^ and abroad.^2,5^

### Clinical characteristics of self-harm

We found that neither the location of occurrence nor the method of self-injury changed during the COVID-19 pandemic. Home remained the most common location for self-harm across all time periods. Cutting continued to be the most common method used; however, there was an increase in more impulsive methods, such as hitting or kicking, during and after the pandemic, suggesting an escalation of aggressive behaviour;^20,30^ this contrasts with the report of Wong et al,^22^ which finds no change in self-harm methods, but also does not include the hitting or kicking as a category. Factors such as impulsivity related to exacerbation of pre-existing mental disorders may have contributed to the use of this method during pandemic-related stress.^31^ Work by De Luca et al^32^ suggests that adolescents who had already self-harmed and had high levels of internalising symptoms and poor regulatory emotional self-efficacy reported higher levels of COVID-19-related stress, putting them at risk for engaging in further self-harm. Also, Shankar et al^21^ reported an increase of 2.29% in visits to the mental health emergency department by children with existing psychiatric diagnoses.

The most commonly injured areas were the face, neck and head; however, such injuries decreased during and after the pandemic. This decline may suggest a preference for less visible forms of self-harm, likely influenced by the presence of family members at home during lockdown. Emotional distress as a consequence of self-harm significantly increased during the pandemic period, potentially reflecting the pandemic’s psychological toll. Although it has been hypothesised that one of the purposes of self-harm is to reduce or distract from negative emotions,^33^ emotional distress may still be present after the period following the release of the negative feelings.

### Clinical management

We observed a notable reduction in hospital admissions during the pandemic, as only severe cases were admitted in order to maintain social distancing measures, thus reducing patient density and potential contagion. In contrast, Ougrin et al^34^ reported that although fewer patients were admitted to the observation ward, no changes were observed for those admitted to the psychiatric in-patient ward; in updates, Wong et al^22^ and McDonnell et al^35^ found no significant change in admissions to any psychiatric ward. In Mexico, during and post-pandemic, out-patient management became the preferred referral choice. At the end of the pandemic, the reopening of services in other institutions likely explains the increase in referrals to external facilities.

### Psychiatric diagnoses

In relation to the main psychiatric diagnosis, the significant rise in affective disorders during and after the pandemic highlights its impact on adolescent mental health. This finding is consistent with other studies evaluating self-harm during the pandemic. For instance, Mayne et al^16^ report an increase from 5 to 6.2% in depressive symptoms among adolescents attending primary care during the pandemic period. Ougrin et al^34^ observed that, among adolescents who self-harmed, the proportion of those with emotional disorders increased from 58 to 66%. The increase in diagnoses such as depression reflects global trends among adolescents, where the pandemic’s uncertainty, fear and prolonged isolation may have led to a surge in mental health problems.^36^ Affective disorders may function as precipitating factors for self-harm, serving as a coping strategy for emotion regulation.^32^ We also observed that some disorders – psychosis, substance misuse and personality disorders – decreased; only the reduction in the last coincides with the observations of Wong et al;^22^ however, any changes in prevalence of these must be considered with caution because of low frequencies.

### Gender differences in prevalence

Regarding the prevalence of self-harm, the trend for females shows multiple fluctuations throughout the pandemic period. Females, particularly in low- and middle-income countries, are more likely to face gender-related challenges, such as domestic responsibilities and gender-based violence,^37^ which may have contributed to greater variability in self-harm prevalence during the early months of the pandemic. Notably, there was a decrease from March to August 2020 among women, coinciding with the early months of the pandemic, while the trend among men consistently declined throughout all pandemic months. Other studies have reported a decrease in self-harm presentations in emergency departments during lockdown,^11,14,38,39^ reflecting reduced access to mental health services during this time. Following the end of the COVID-19 pandemic (April 2023), we observed a sharp increase in self-harm cases among both male and female patients. This result is striking; we would expect that the cessation of restrictive measures and full reintegration into school and social activities would lead to improved emotional well-being for adolescents, but the findings suggest the opposite. Gracia et al^40^ in Spain, who noted an increase in suicide attempts among girls after resuming school, reported equivalent results between September 2020 and March 2021. Wong et al’s study,^22^ which analysed patient records from 32 countries, including Mexico, also reported a doubling in the rate of self-harm presentations from March to April 2021. Additionally, Bruns et al^14^ found a doubling of the suicide attempt ratio in a German sample. The present study covers a longer evaluation period.

### Long-term effects of the pandemic

The upward trend in the prevalence of self-harm points to the long-term effects of pandemic stress on adolescent mental health. Returning to a ‘new normal’ after such an extended period of social isolation presented new challenges, including adapting to new classmates, transitioning to higher school levels while missing previous in-person grades, increased academic load and re-establishing daily routines (e.g. earlier wake-up times, hygiene practices, etc.). It is likely that aspects of the return to normalcy served as stressors for adolescents, especially those with pre-existing mental health conditions. Concurrently, as mental health services became more accessible again, young people had greater opportunities to receive care.

This is one of the first studies to address post-pandemic trends in self-harm presentations to psychiatric emergency departments, as most of the studies, even those that include uninterrupted time periods, only address the pre- and pandemic periods up to 2021. Although the pandemic may be over, its repercussions on mental health may continue for a prolonged time.

### Limitations

The results of this study must be interpreted within some limitations. This is an observational and retrospective study: we cannot establish causality with the COVID-19 pandemic. Additionally, this study was conducted at a single psychiatric hospital in Mexico City, which may limit the generalisability of the findings to other regions of Mexico or to other countries with different healthcare systems. Finally, the reliance on emergency department data may not fully capture the prevalence and characteristics of self-harm in the broader adolescent population as, for instance, motive of self-injury was not recorded.

## Data Availability

The data that support the findings of this study, along with the code for replicating the regression analysis, are openly available from FigShare at https://doi.org/10.6084/m9.figshare.26928694.v1. Certified Professionals in Autism Detection and Diagnosis (www.procedda.com).
